# Cause-specific telomere factors deregulation in hepatocellular carcinoma

**DOI:** 10.1186/1756-9966-32-64

**Published:** 2013-09-11

**Authors:** Manale El Idrissi, Valérie Hervieu, Philippe Merle, Franck Mortreux, Eric Wattel

**Affiliations:** 1 UMR5239 Oncovirologie et Biothérapies, Faculté de Médecine Lyon Sud, ENS – HCL, Université Lyon 1, CNRS, Pierre Bénite, France; 2 Service Central d’Anatomie et Cytologie Pathologiques, Hospices Civils de Lyon, Université Lyon 1, Hôpital Edouard Herriot, 69437, Cedex 03 Lyon, France; 3 INSERM U1052, CNRS UMR5286, Centre de Recherche en Cancérologie de Lyon, F69008 Lyon, France; 4 Université Lyon-1, F69622 Villeurbanne, France; 5 Hospices Civils de Lyon, Service d’Hépatologie et de Gastroentérologie, Groupement Hospitalier Lyon Nord, F69000 Lyon, France; 6 Service d’Hématologie, Pavillon Marcel Bérard, Centre Hospitalier Lyon-Sud, Université Lyon I, 165, chemin du Grand Revoyet, 69495 Pierre Bénite, France

**Keywords:** Liver, Hepatocellular carcinoma, Telomere, Telomerase, Shelterin, Hepatitis B virus, Hepatitis C virus, Alcohol, Cirrhosis

## Abstract

**Background:**

Among the numerous genetic defects associated with hepatocarcinogenesis, telomere abnormalities appear to play a role both in tumor promotion and maintenance. Telomeres, the chromosome extremities, are protected by specific proteins, the shelterin complex and by additional factors. Besides telomerase dysregulation, expression changes of these telomere factors have been observed in cancers.

**Methods:**

Here, we tested the hypothesis that such dysregulation might occur in hepatocellular carcinoma (HCC) with specific patterns depending on the cause of HCC. We compared telomere length, telomerase activity (TA), *hTERT* and telomere genes expression using PCR and Western-blot analyses between non-cirrhotic liver, peritumoral cirrhotic tissue (40 samples) and cancerous tissue (40 samples) derived from 40 patients with HBV-, HCV-, or alcohol-related HCC.

**Results:**

Alterations in TA, *hTERT* expression and telomere length between non-cirrhotic, cirrhotic, and tumor samples were not significantly influenced by the cause of HCC. In contrast, the expression pattern of *hTR*, shelterin, and non-shelterin telomere protective factors clearly distinguished the 3 causes of cirrhosis and HCC. For patients with HBV diseased liver, when compared with non-cirrhotic liver, the cirrhotic tissue underexpressed all shelterin and all but *HMRE11A* and *RAD50* non-shelterin telomere factors. For HCV the expression level of *POT1*, *RAP1*, *Ku80*, and *RAD50* was higher in cirrhotic than in non-cirrhotic liver samples without evidence for significant transcriptional change for the remaining genes. For alcohol-related liver diseases, the expression level of *POT1*, *RAP1*, *TIN2*, *hMRE11A*, *hMRE11B*, *Ku70*, *Ku80*, *RAD50*, *TANK1*, and *PINX1* was higher in cirrhotic than in non-cirrhotic liver samples. For the 3 causes of HCC, there was no significant change in shelterin and non-shelterin gene expression between cirrhosis and HCC samples.

**Conclusions:**

These results validate our hypotheses and demonstrate that cirrhosis and HCC add-up numerous telomere dysfunctions including numerous cause-specific changes that appear to occur early during the course of the disease.

## Background

Hepatitis B (HBV) or C virus (HCV) infection and alcohol consumption are leading causes of hepatocellular carcinoma (HCC) that predominantly develops from chronic hepatitis and cirrhosis [1]. Among the numerous genetic and epigenetic defects associated with carcinogenesis [2], telomere abnormalities play a role in tumor promotion and maintenance [3-9]. Telomeres, the chromosome extremities, are elongated by the human telomerase, the catalytic moiety of which is encoded by the human telomerase reverse transcriptase (*hTERT*) gene [10]. Additionally, telomeres are protected by specific proteins, the shelterin complex [11] and by additional non-specific factors such as human meiotic recombination 11 homolog A and B (hMRE11A and B), Ku proteins 70 and 80 (Ku70 and Ku80), Nijmegen breakage syndrome-1 (NBS1), RAD50, tankyrase 1 and 2 (TANK1 and 2), Werner syndrome helicase (WRN), and PIN2/TRF1-interacting, telomerase inhibitor 1 (PINX1) [12]. These factors prevent telomere degradation and facilitate telomerase-based telomere elongation.

Short or unprotected telomeres are recombinogenic and can therefore promote tumorigenesis [3]. In normal cells, dysfunctional telomeres trigger the DNA damage response and replicative cellular senescence [10,13-18]. Early oncogenic events frequently involve evasion of the DNA damage response, which allows the clonal persistence of cells bearing a telomere-associated genetic instability. During early tumor development, *hTERT* is frequently expressed and allows the clone to bypass mitotic catastrophe and replicative senescence, contributing to malignant immortalization [4,5,19-21]. Therefore, impaired telomere protection and/or elongation represent putative oncogenic events. Indeed, numerous oncogenes or tumor suppressor genes have been reported to interfere with the telomere machinery. In the liver, telomere shortening correlates with chromosomal instability and the development of HCC [4,6,8]. Hepatotropic viruses and alcohol have been reported to interfere with telomere homeostasis. For example, *hTERT* transcription was found to be activated upon HBV DNA integration in the vicinity of the *hTERT* gene [22] while HBV encoded X (HBx) [23-27] or preS2 [28,29] proteins promote *hTERT* expression and contributed to clonal persistence. However, some mutated HBx have been reported to possess repressive effects on *hTERT* transcription [25]. The HCV core protein has been demonstrated to enhance telomerase activity [30] while alcohol exposure triggers premature senescence with accelerated telomere shortening [31].

Changes in telomere length, telomerase activity and *hTERT* expression have been extensively explored at different steps of hepatocarcinogenesis. However, to our knowledge, the status of shelterin and non-shelterin telomere factors has not been examined during liver carcinogenesis. Furthermore, little is known about the interactions between telomere alterations and the cause of HCC, although hepatitis viruses and alcohol are known to possess specific and distinct effect on telomere homeostasis *in vitro*[22,23,25-29,31]. Dissecting telomere factors’ deregulation during carcinogenesis has revealed novel oncogenic pathways, prognostic markers, and therapeutic targets aimed at preventing or treating cancers. The aim of this study was to determine whether the main causes of HCC might trigger distinct telomere effects *in vivo*.

## Methods

### Samples

The medical ethics committee of the Hospices Civils de Lyon approved the study, and the informed consent was obtained from patients in accordance with the Declaration of Helsinki and with institutional guidelines. The study population consisted of paired tumor and non-tumor tissues from 40 patients suffering from HCC including 10 HBV-, 10 HCV-, 10 alcohol-related and 10 HCC without HCV viral or alcohol exposure (idiopathic HCC). Serological markers for HBV (HBsAg, HBeAg and anti-HBe), HCV (anti-HCV) were tested in all cases by commercial enzyme immunoassays. The clinical and histological data accompanying the samples analyzed are shown in Table 1. Tissue samples from patients resected for HCC were collected during surgery and divided into two parts: one was immediately cut into small pieces, snap-frozen in liquid nitrogen and stored in deep freezer; the other was fixed in 10% formalin and paraffin-embedded for histopathological examination and immunohistochemistry. Histological analysis and immunohistochemistry were performed at the Department of Pathology at the Hospices Civils de Lyon.

**Table 1:** Clinical features of the 40 patients with hepatocellular carcinoma

**UPN**	**Age**	**Sex**	**Serological markers**		**Peritumoral histology**
			**HBV**	**HCV**	
***HBV-associated hepatocellular carcinoma***					
1	65	M	+	-	Cirrhotic
2	58	M	+	-	Cirrhotic
3	35	F	+	-	Normal
4	47	M	+	-	Normal
5	58	M	+	-	Cirrhotic
6	50	F	+	-	Cirrhotic
7	60	M	+	-	Cirrhotic
8	33	M	+	-	Cirrhotic
9	74	M	+	-	Cirrhotic
10	44	M	+	-	Cirrhotic
***HCV-associated hepatocellular carcinoma***					
11	56	M	-	+	Cirrhotic
12	68	M	-	+	Cirrhotic
13	58	M	-	+	Cirrhotic
14	61	F	-	+	Cirrhotic
15	55	M	-	+	Cirrhotic
16	57	M	-	+	Cirrhotic
17	73	M	-	+	Cirrhotic
18	70	M	-	+	Cirrhotic
19	47	M	-	+	Normal
20	66	M	-	+	Cirrhotic
***Alcohol-associated hepatocellular carcinoma***					
21	49	F	-	-	Cirrhotic
22	56	M	-	-	Cirrhotic
23	49	M	-	-	Cirrhotic
24	83	M	-	-	Cirrhotic
25	59	M	-	-	Cirrhotic
26	58	M	-	-	Cirrhotic
27	62	F	-	-	Cirrhotic
28	58	M	-	-	Cirrhotic
29	63	M	-	-	Cirrhotic
30	59	M	-	-	Cirrhotic
***Idiopathic hepatocellular carcinoma***					
31	45	M	-	-	Normal
32	66	M	-	-	Normal
33	72	F	-	-	Normal
34	72	M	-	-	Normal
35	75	F	-	-	Normal
36	76	M	-	-	Cirrhotic
37	35	M	-	-	Normal
38	67	M	-	-	Normal
39	72	F	-	-	Normal
40	78	M	-	-	Normal

### Telomere length assay

Each sample was homogenized and total cellular DNA was extracted using phenol chloroform. The average telomere length was measured in all samples using the TeloTAGGG Telomere length Assay (Roche). Briefly, purified genomic DNA (6–8 μg) was digested by specific restriction enzymes. The DNA fragments were separated by gel electrophoresis and transferred to a nylon membrane using Southern blotting. The blotted DNA fragments were hybridized to a digoxigenin-labeled probe specific to telomere repeats and incubated with a digoxigenin-specific antibody coupled to alkaline phosphate. Finally, the immobilized probe was visualized by a sensitive chemiluminescence substrate and the average TRF length was assessed by comparing the signals relative to a molecular weight standard.

### Quantification of telomerase activity

The telomeric repeats amplification protocol (TRAP) was combined with real-time detection of amplification products to determine telomerase activity using a Quantitative Telomerase Detection kit (US Biomax) following the manufacturer’s recommendations. Total protein extracts (0.5 μg) were used for each reaction. The end products were resolved by PAGE on a 12.5% non-denaturing gel, stained with Sybr Green Nucleic Acid gel stain (Invitrogen) and visualized using the Bio-Rad Molecular Imager ChemiDoc System.

### Real-time quantitative reverse transcriptase-polymerase chain reaction (PCR)

Each tissue sample was homogenized and total cellular RNA was extracted using the MasterPure Complete DNA and RNA Purification Kit (Epicentre) according to the manufacturer’s instructions. Before reverse transcription, RNA was treated with DNase (Invitrogen-Life technology) to prevent DNA contamination. First-strand complementary DNA (cDNA) was synthesized from 0.5 μg RNA using random primers (Promega) and Superscript II reverse transcriptase (Invitrogen). The RNA concentration and purity were determined using a NanoDrop instrument (Thermo Scientific). The primer sequences are available upon request. Primer sets used to quantify gene expression were first tested in PCR with a control cDNA to ensure specific amplification, as evidenced by the presence of a unique specific signal after agarose gel electrophoresis. PCR assays were performed on an ABI Prism 7000 sequence detection system (Applied Biosystems) using 5 μL of cDNA, 6 μL of SYBR Green Master Mix, 0.25 μL of ROX (Invitrogen) and 0.75 μL of primers at 10 μM. Thermal cycling consisted of a first cycle at 50°C for 2 min and 95°C for 10 min, followed by 40 cycles at 95°C for 15 seconds and 60°C for 1 min. Finally at the end of each PCR run, temperature was raised up to 95°C in order to check the melting curve. The expression of each gene of interest was normalized against 2 housekeeping genes, Gus (NM_000181) and ERCC1 (NM_001983), which have been validated using the BestKeeper software tool [32] to adjust for variations in RNA amount and cDNA synthesis. The relative quantification was depicted as the fold-change in expression of each gene using the formula 2ΔΔCt, as previously described [33]. Each assay was performed in duplicate.

### Western blot analysis

The antibodies to MRE11 were purchased from Santa Cruz Biotechnology (Sc-22,767), hTERT (ab-32,020) and POT1 (ab-124,784) were purchased from Abcam, TRF2 (05-521) was purchased from Millipore, and the antibodies to Ku80 (2753S) and beta-Actin (4967S) were purchased from Cell Signaling Technology. Tumor extracts were homogenized and then lysed. The protein concentration was determined using the Bio-rad D_c_ Protein Assay Kit (Bio-rad). Equal amounts of proteins were subjected to sodium dodecyl sulfate 10% polyacrylamide gel electrophoresis (MiniProtean TGX, BioRad) and fractionated proteins were transferred to PVDF membranes (Transblot Turbo, BioRad). These membranes were blocked in TBS containing 5% nonfat milk, 0.05% Tween 20, and then probed with the appropriate antibody followed by incubation with a secondary IgG HRP-linked antibody (Cell Signaling Technology). The blots were then developed using an enhanced chemiluminescence detection system (Clarity Western ECL, BioRad).

### Immunocytochemistry

Ki67 was assessed using the anti-Ki67 MoAbs (clone MIB-1 Dako, on BenchMark Ventana XT). CC1 treatment (1/50) was performed before ultraview revelation. Ki67 immunohistochemistry was quantified by a pathologist. The percentage of labeled nuclear area over the total neoplastic and the non-neoplastic nuclear area in the section was quantified from 2000 cells in areas of highest nuclear labeling.

### Statistical analysis

Statistical analysis was performed using the 2-tailed Student’s t test or the Mann–Whitney U rank sum test. P < 0.05 was considered statistically significant in all analyses. All data analyses were performed using SPSS statistical software version 20.

## Results

**Figure 1:**
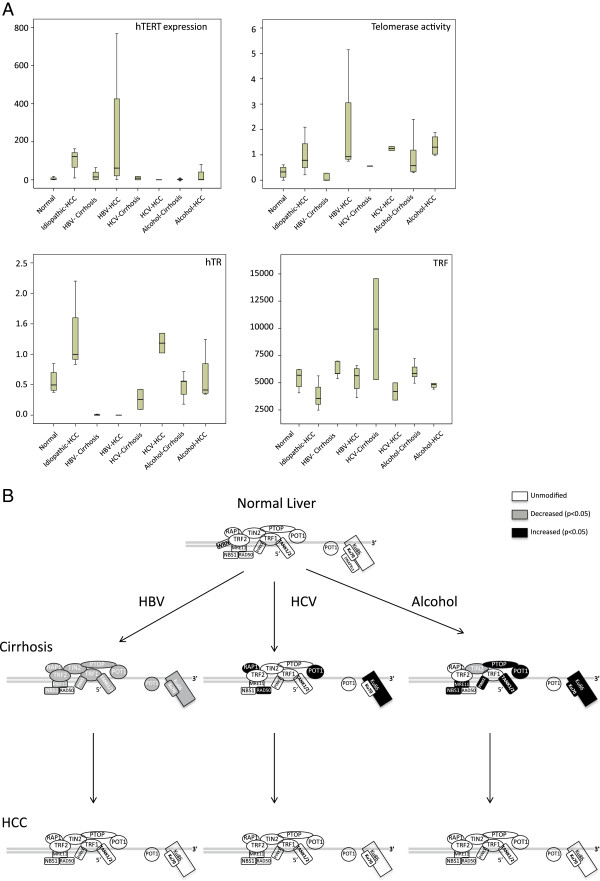
**Common and specific telomere abnormalities between HBV-, HCV-, and alcohol-associated cirrhosis and hepatocellular carcinoma. A**. Distribution of *hTERT* and *hTER* expression, telomerase activity and TRF length among the main causes of hepatocellular carcinoma. **B**. Alteration in shelterin and non-shelterin gene expression at the two main steps of liver carcinogenesis *in vivo*. Significantly overexpressed genes (p < 0.05, Mann Whitney test) are represented in black whereas significantly underexpressed genes are represented in gray.

**Figure 2:**
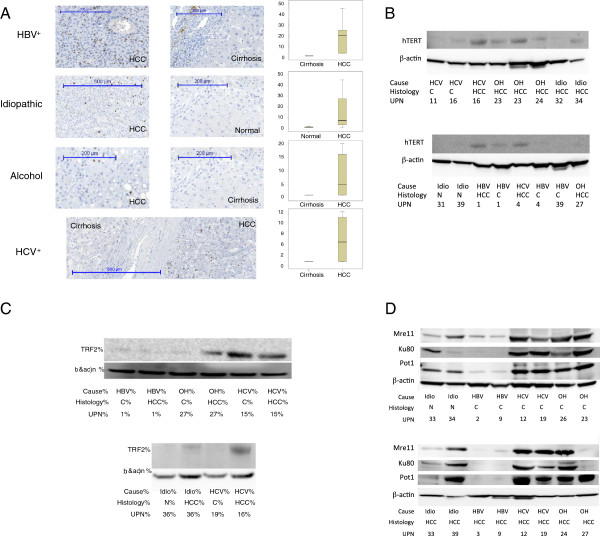
**Immunohistochemistry and Western-blot analysis. (A)** Ki67, **(B)** hTERT, **(C,D)** shelterin and non-shelterin and **(D)** telomere factors in the main causes of cirrhosis and hepatocellular carcinoma.

The main objective of this study was to determine whether differences exist in telomere deregulation between HBV-, HCV-, and alcohol-associated liver carcinogenesis. Liver carcinogenesis is a multistep process where clinical and histopathological features frequently permits the differentiation of the two main phases that include a cirrhotic stage followed by the development of overt HCC. Our collection of 80 liver samples was obtained from 40 patients with HCC. For each case 2 samples were analyzed that corresponded to tumoral and peritumoral tissue. The Table 1 shows that in 12 cases of HCC, peritumoral samples corresponded to histologically normal, non-cirrhotic liver tissue whereas in the 28 remaining cases, the peritumoral tissue was cirrhotic. We assumed that the development of cirrhosis from a histologically non-cirrhotic liver represents an early event during liver carcinogenesis, whereas the development of HCC from a cirrhotic liver reflects later carcinogenic events. Accordingly, in order to assess telomere dysregulation at the early and late stages of liver carcinogenesis, we compared cell proliferation, Telomere Restriction Fragment (TRF) length, TA, *hTERT*, human telomerase RNA compound (*hTR)*, shelterin and non-shelterin telomere factor expression between non-cirrhotic and cirrhotic liver samples and between cirrhotic and HCC liver samples for each cause of HCC (Additional file 1: Table S1, Additional file 2: Table S2. Figure 1 represents the distribution of TRF length, *hTERT* and *hTR* expression, TA (Figure 1A) and telomere factors expression (Figure 1B) in peritumoral and tumoral samples derived from patients suffering from idiopathic, HBV-, HCV-, and alcohol-related HCC. Figure 2 represents the expression of Ki67 (Figure 2A), *hTERT* (Figure 2B) and telomere protective factors (Figure 2B and C) at the protein level.

### Telomere deregulation at the early stage of HBV-associated hepatocarcinogenesis

Expression of the proliferative marker Ki67 was not significantly different between the 8 HBV positive cirrhotic samples and the 12 non-cirrhotic liver samples deriving from patients with HCC. As illustrated in Figure 1A, the level of *hTERT* expression was significantly higher in the 8 HBV positive cirrhotic samples than in the 12 non-cirrhotic liver samples (p = 0.040, Mann–Whitney test). In contrast, there was no significant difference in the level of TA between the cirrhotic and non-cirrhotic sample categories. HBV-associated cirrhosis expressed significantly lower *hTR* levels when compared to histologically non-cirrhotic liver tissue: 0.0053 versus 0.3574 arbitrary units (p < 10^-4^, Mann–Whitney test) (Figure 1A). The TRF length was longer in HBV positive cirrhotic samples than in non-cirrhotic samples (6.60 kbp versus 5.69 kbp) but the difference was not statistically significant. Comparative Western-blot analysis of hTERT expression in HBV positive cirrhotic samples versus non-cirrhotic liver samples confirmed the qRTPCR results for *hTERT* expression (Figure 2B). Table 2 and Figure 1B show that all shelterin and non-shelterin telomere factors except *HMRE11A* and *RAD50* were significantly underexpressed in HBV positive peritumoral cirrhotic samples. Comparative Western-blot analysis confirmed that protection of telomeres 1 (POT1), telomere repeat factor 2 (TRF2), HMR11A/B, and Ku80 had lower expression levels in HBV positive cirrhotic samples than in non-cirrhotic liver samples (Figure 2C and D). These results suggest that at the telomere level, the development of HBV-associated cirrhosis includes strong *hTERT* overexpression and considerable repression of *hTR*, shelterin, and non-shelterin telomere factors. Similar results were obtained when the 8 HBV^+^ cirrhotic samples were compared with the 9 non-cirrhotic liver samples derived from patients with idiopathic HCC (data not shown).

**Table 2:** Cause-specific differences in telomeric gene expression between cirrhotic and non-cirrhotic liver samples

	**Non-cirrhotic**	**Cirrhotic**			***p***		
	**(n = 12)**	**HBV (n = 8)**	**HCV (n = 9)**	**Alcohol (n = 10)**	***For HBV***	***For HCV***	***For alcohol***
***Shelterin***							
*POT1*	0.0021	0.0000	0.0125	0.0090	0.0480	0.0100	0.0050
*PTOP*	0.0094	0.0000	0.0037	0.0055	0.0200	ns	ns
*RAP1*	0.1570	0.0016	0.4210	0.4091	0.0070	0.0080	0.0060
*TIN2*	0.3510	0.0018	0.0510	0.0804	0.0010	ns	<10-4
*TRF1*	0.5585	0.0117	0.2271	0.2488	<10-4	ns	ns
*TRF2*	0.0016	0.0000	0.0016	0.0012	0.0050	ns	ns
***Non-Shelterin***							
*HMRE11A*	0.0187	0.0006	0.0627	0.0764	ns	ns	0.0070
*HMRE11B*	0.0359	0.0008	0.0492	0.0886	0.0030	ns	0.0020
*Ku70*	0.0955	0.0045	0.1704	0.1825	<10-4	ns	0.0440
*Ku80*	0.0408	0.0033	0.1209	0.1316	0.0200	0.0290	0.0120
*NBS1*	0.0266	0.0002	0.0304	0.0403	0.0030	ns	ns
*RAD50*	0.0030	0.0002	0.0091	0.0108	ns	0.0180	0.0500
*TANK1*	0.0468	0.0005	0.0788	0.0945	<10-4	ns	0.0030
*TANK2*	0.0129	0.0000	0.0188	0.0127	0.0200	ns	ns
*Pinx1*	0.0131	0.0001	0.0083	0.0219	0.0020	ns	0.0210

### Telomere deregulation at the early stage of HCV-associated hepatocarcinogenesis

Expression of the Ki67 proliferation marker was not significantly different between the 9 HCV positive cirrhotic samples and the 12 non-cirrhotic liver samples deriving from patients with HCC. There was no significant difference in the expression level of TA, *hTERT* and *hTR* between the two sample categories (Figure 1A). Western-blot analysis of hTERT expression confirmed the qRTPCR results for *hTERT* expression (Figure 2B). Shelterin, *POT1* and repressor-activator protein 1 (*RAP1)* were demonstrated to be significantly overexpressed in HCV positive cirrhotic samples when compared with non-cirrhotic liver samples. The remaining factors displayed an identical (*TRF2*) or a non-significant reduced expression level (Table 2). In contrast to HBV, all telomere factors except *Pinx1* non-shelterin were overexpressed in cirrhotic peritumoral HCV positive samples, as compared to non-cirrhotic liver samples (Figure 1C, Table 2). Indeed, the expression of *Ku80* (p = 0.029) and *RAD50* (p = 0.018) was approximately 3 times higher than that of the control samples. Western-blots confirmed that POT1, HMRE11A/B, and KU80 were more expressed in HCV positive cirrhotic samples than in non-cirrhotic liver samples (Figure 2D). These results suggested that at the telomere level, the main changes that accompany the development of HCV-associated cirrhosis predominately involve the overexpression of *POT1*, *RAP1*, *Ku80*, and *RAD50* telomere factors.

### Telomere deregulation at the early stage of alcohol-associated hepatocarcinogenesis

Expression of the Ki67 proliferative marker was not significantly different between alcohol-associated cirrhotic and non-cirrhotic liver tissues deriving from patients with HCC. There was no significant difference in TRF length, TA, *hTERT* and *hTR* expression between the two sample categories (Figure 1A). Western-blot analysis of hTERT expression confirmed the qRTPCR results (Figure 2B). Shelterin, *POT1* (p = 0.005) and *RAP1* (p = 0.006) were demonstrated to be significantly overexpressed in alcohol-associated cirrhotic tissues, whereas other shelterins were found to be underexpressed, with TRF1-interacting nuclear protein 2 gene *(TIN2)* showing a significant difference (Table 2). All non-shelterin telomere factors, except *TANK2* and *Pinx1*, contained a transcriptional pattern that resembled that in HCV cirrhotic samples. Accordingly, all telomere factors except the *TANK2* non-shelterin were overexpressed in cirrhotic alcohol-exposed liver with significant differences demonstrated for *HMRE11A*, *HMRE11B*, *Ku70*, *Ku80*, *RAD50*, *TANK1*, and *Pinx1* (Table 2, Figure 1C). Western-blot analyses confirmed the qRTPCR results for POT1, TRF2, HMR11A/B, and KU80 (Figure 2C and D). These results suggested that at the telomere level, the main changes accompanying the development of alcohol-associated cirrhosis and fibrosis predominantly involve the overexpression of *POT1*, *RAP1*, *HMRE11A*, *HMRE11B*, *Ku70*, *Ku80*, *RAD50*, *TANK1*, and *Pinx1* telomere factors. Taken together, these results indicate that the development of HBV-, HCV-, and alcohol-related cirrhosis rely on clearly distinct telomere perturbations and suggests that these distinct carcinogens possess specific effects on telomere homeostasis. Consequently, 3 kinds of cirrhotic tissues displayed significant differences in the expression of telomere factors (Figure 1, Additional file 3: Table S3).

### Telomere deregulation at the late stage of HBV-associated hepatocarcinogenesis

Having demonstrated the cause-specific changes in telomere factors’ expression between cirrhotic and non-cirrhotic livers, *i.e*. during early hepatocarcinogenesis, we next sought to investigate whether these differences persist at the late stages of HCC development. To this end we compared telomere deregulations between cirrhotic and tumoral samples deriving from patients with HCC. We first compared the 10 HBV-associated HCC samples with their 8 cirrhotic peritumoral samples. Expression of the Ki67 proliferative marker was significantly increased in HBV-associated HCC, as compared with HBV-associated cirrhosis (p = 0.002, Mann–Whitney test). The TRF length was significantly shorter in tumor samples than in cirrhotic samples (p = 0.05, Mann–Whitney test) whereas the levels of TA and *hTERT* expression were significantly higher in HBV positive HCC (p = 0.017 for hTERT and p = 0.002 for TA, Mann–Whitney test) without any significant difference in the level of *hTR* expression between the 2 tissues (Figure 1A). Western-blotting analyses confirmed the qRTPCR results for *hTERT* expression (Figure 2B). Table 3 shows that with the exception of *Pinx1*, where there was a trend for higher expression in HCC, all shelterin and non-shelterin genes remained underexpressed in HBV positive HCC without any significant difference between cirrhosis and HCC. Western-blot analysis of TRF2, HMRE11A/B, Ku80, and POT1 confirmed the qRTPCR results (Figure 2C and D). These results suggested that at the telomere level, augmented TA and *hTERT* expression represent the major significant telomere deregulation distinguishing HBV-associated HCC from HBV-associated cirrhosis. Accordingly, comparison of HBV-related HCC with non-cirrhotic liver samples demonstrated similar differences as the comparison of HBV-related cirrhosis with non-cirrhotic liver samples (Additional file 4: Table S4).

**Table 3:** Cause-specific differences in telomeric gene expression between cirrhotic/fibrotic and HCC tissue samples

	**HBV**			**HCV**			**Alcohol**		
	**Cirrhotic and/or Fibrotic (n = 8)**	**HCC (n = 10)**	***p***	**Cirrhotic and/or Fibrotic (n = 9)**	**HCC (n = 10)**	***p***	**Cirrhotic and/or Fibrotic (n = 10)**	**HCC (n = 10)**	***p***
***Shelterin***									
*POT1*	0.0000	0.0000	ns	0.0125	0.0203	ns	0.0090	0.0060	ns
*PTOP*	0.0000	0.0000	ns	0.0037	0.0064	ns	0.0055	0.0071	ns
*RAP1*	0.0016	0.0000	ns	0.4210	0.5059	ns	0.4091	0.2538	ns
*TIN2*	0.0018	0.0033	ns	0.0510	0.0581	ns	0.0804	0.0876	ns
*TRF1*	0.0117	0.0209	ns	0.2271	0.1626	ns	0.2488	0.2886	ns
*TRF2*	0.0000	0.0000	ns	0.0061	0.0015	ns	0.0012	0.0012	ns
***Non Shelterin***									
*HMRE11A*	0.0006	0.0000	ns	0.0627	0.0811	ns	0.0764	0.0536	ns
*HMRE11B*	0.0008	0.0000	ns	0.0492	0.0508	ns	0.0886	0.0850	ns
*Ku70*	0.0045	0.0024	ns	0.1704	0.2418	ns	0.1825	0.1645	ns
*Ku80*	0.0033	0.0015	ns	0.1209	0.1494	ns	0.1316	0.0853	ns
*NBS1*	0.0002	0.0024	ns	0.0304	0.0317	ns	0.0403	0.0501	ns
*RAD50*	0.0002	0.0000	ns	0.0091	0.0118	ns	0.0108	0.0101	ns
*TANK1*	0.0005	0.0000	ns	0.0788	0.0761	ns	0.0945	0.0869	ns
*TANK2*	0.0000	0.0006	ns	0.0188	0.0255	ns	0.0127	0.0171	ns
*Pinx1*	0.0001	0.0049	ns (0.054)	0.0083	0.0107	ns	0.0219	0.0165	ns

### Telomere deregulation at the late stage of HCV-associated hepatocarcinogenesis

HCV-associated HCC expressed higher levels of the Ki67 proliferative marker (6% versus 1%) than peritumoral cirrhotic tissue samples but the difference was not statistically significant. When compared to their peritumoral cirrhotic tissue samples, HCV positive HCC expressed higher amounts of *hTERT* transcripts (p = 0.54) and *hTR* (p = 0.021) and they displayed increased TA (p = 0.036) when compared with HCV positive cirrhosis (Figure 1A). The TRF length was shorter in HCV-associated cirrhosis than in HCC but the difference was not statistically significant (5.1 kbp versus 6.6 kbp, p = 0.39) (Figure 1A). Table 3 shows that the pattern of shelterin and non-shelterin genes expression was not significantly different between HCV-associated HCC and HCV-associated cirrhosis. Western-blot analysis confirmed qRTPCR results (Figure 2B,C, and D). These results suggested that at the telomere level, increased TA and *hTR* expression represent the major significant telomere deregulation that distinguishes HCV-associated HCC from HCV-associated cirrhosis.

### Telomere deregulation at the late stage of alcohol-associated hepatocarcinogenesis

When compared to their peritumoral cirrhotic tissue samples, alcohol-associated HCC expressed higher levels of the Ki67 proliferative marker (8% versus 1%) but the difference was not statistically significant. Figure 1A shows that TA, *hTERT* and *hTR* expressions were augmented in alcohol-associated HCC but these differences were not statistically significant. Table 3 shows that the pattern of shelterin and non-shelterin genes expression was not significantly different between alcohol-associated HCC and alcohol-associated cirrhosis. Western-blot analysis confirmed the qRTPCR results (Figure 2C and D). These results suggested that at the telomere level, there is no significant deregulation that distinguishes alcohol-associated HCC from alcohol-associated cirrhosis.

## Discussion

The data suggest that the development of HCC involves the accumulation of numerous telomere dysfunctions that appear to include cause-specific deregulations. Our sample collection permitted the comparison of histologically non-cirrhotic livers with cirrhosis and HCC in the context of HBV and HCV infections, and alcohol exposure. Given that HCC mostly develop from cirrhotic livers, we assumed that comparing histologically non-cirrhotic liver samples with cirrhotic liver samples would reflect early carcinogenesis whereas comparing cirrhotic liver samples with tumor samples would reflect later carcinogenic events. Indeed, alterations in TRF length, TA, *hTERT* and *hTR* expression were identified at both the early and late steps of hepatocarcinogenesis. These alterations were observed roughly in parallel among the 3 different causes of HCC. In contrast, the numerous changes demonstrated in the expression of telomere protective factors appeared to be restricted to early hepatocarcinogenesis. Additionally, these changes permitted the identification of a gene expression signature for each cause of cirrhosis and HCC. There was furthermore, evidence that the telomere phenotype of HBV-associated-cirrhosis and HCC was different from that of the other causes of cirrhosis and HCC.

No correlation was found between TA, *hTERT* expression and telomere length with respect to the cause of cirrhosis and HCC. This result is in agreement with the study of Saini *et al*. who compared TA, TRF and *hTERT* expression between HBV, HCV, and non-B non-C-related HCC [34]. In contrast, Guo *et al*. reported that HbsAg positive HCC expressed higher amounts of *hTERT* mRNA than HbsAg negative HCC [35]. Whatever the cause, there was no significant difference in TRF length between cirrhotic and non-cirrhotic samples. This result was in agreement with the lack of difference in cell proliferation between the 2 samples categories, as assessed by the quantification of Ki67 expression. In contrast, the tumor samples expressed higher levels of the Ki67 proliferative marker and contained shorter telomeres than either non-cirrhotic or cirrhotic samples. There was no precise correlation between the level of *hTERT* expression measured by qRTPCR and the level of TA measured by the quantitative TRAP assay, suggesting that posttranscriptional modifications might participate to modulate TA during hepatocarcinogenesis. Additionally, there was no significant correlation between either *hTERT* expression or TA and telomere length. Conversely, Figure 1A shows that the shorter were the telomeres in sample sets, the higher were TA and *hTERT* expression in these samples. This conflicting data might be explained, at least in part, by changes in regulating access of the telomere to the telomerase in liver cells, *i.e*. by changes in telomere proteins content.

Accumulating evidence suggests that telomeric factors dysregulation is involved in cancer development as has been demonstrated in the maintenance of the tumor phenotype. To our knowledge, this study is the first which investigates the expression of the main telomere protective genes in the main subtype of cirrhosis and HCC. Previously, Oh *et al*. demonstrated that expression of TRF1, TRF2 and TIN2 was gradually increased according to the progression of hepatocarcinogenesis in HBsAg positive individuals [36]. In this study, HBV-, HCV- and alcohol-associated cirrhosis displayed significantly different distinct patterns of telomere protective factor expression, as compared with that of non-cirrhotic liver (Table 2). The 3 subtypes of cirrhosis possessed a specific signature, with respect to telomere protective factor expression (Additional file 3: Table S3). Although the expression level of all the shelterin and non-shelterin telomere factors was not equally distributed between the 3 causes of cirrhosis (Additional file 3: Table S3), the telomere phenotype of HBV-associated-cirrhosis appeared different from that of the 2 other causes of cirrhosis. When compared with non-cirrhotic liver, HBV-associated cirrhosis displayed a dramatic repression of all shelterin and non-shelterin factors except HMRE11A and RAD50. In contrast, the alterations in telomere factor expression between non-cirrhotic and cirrhotic samples were similar between HCV- and alcohol-associated cirrhosis. Accordingly, the expression pattern of all telomere factors, except TIN2 and HMRE11B, was identical between HCV- and alcohol-associated cirrhosis (Additional file 3: Table S3). These results suggest that cause-specific factors are involved in initiating telomere dysfunction in the liver. For example, HBV-associated cirrhosis displayed very low amounts of TRF2 that has been demonstrated to elicit telomere shortening *ex vivo*[37].

Whatever the cause, the levels of shelterin and non-shelterin telomere factors expression were not significantly different between cirrhotic and HCC samples (Figure 1B and Table 3). Again, the expression pattern of telomere protective factor of HBV-associated-HCC remained distant from that of the 2 other causes of HCC, which closely resembled that of idiopathic HCC (Additional file 5: Table S5). This suggests that in HCC, the cause-specific expression pattern of shelterin and non-shelterin factors has been acquired early during the course of the disease. Given that these factors are thought to prevent proper telomerase-telomere interaction, the present results partly explains the combination of high TA with short telomeres in HCC.

## Conclusion

In conclusion, the control of telomere homeostasis is significantly dysregulated during liver carcinogenesis and each cause of cirrhosis and HCC includes specific dysregulation of telomere protective factors. These changes occur early, at the cirrhotic stage, and persist to the tumor stage, which suggests that they contribute to both tumor development and tumor progression. By demonstrating gene and protein dysregulation that are thought to prevent proper telomerase-telomere interactions, the present results partly explain the combination of high TA with short telomeres in HCC. Shortened and deprotected telomeres are recombinogenic and contribute to the genetic instability that characterize HCC and facilitate tumor progression, tumor recurrence and resistance to treatment [5-8,10]. Importantly, hepatocytes have been reported to tolerate telomere dysfunctions [37], reinforcing the tumorigenic impact of alcohol-, HBV-, and HCV-associated telomere damage in exposed individuals. Targeting telomerase is becoming a promising approach for the treatment of HCC [38-40] and our present results also support such an approach for treating the main causes of this disease. In contrast, our results suggest that targeting the cause-specific deregulation of telomere protective factors might be of interest in the prevention or the treatment of cirrhosis and HCC.

## Competing interests

The authors declare that they have no competing interests.

## Authors’ contributions

MEI carried out the most experimental work. VH performed the sample collection and Ki67 assays. PM performed the sample collections, provided clinical data. PM, FM, and EW were responsible for the design of the study and its coordination. PM, EW, and FM wrote the manuscript. All authors read and approved the final manuscript.

## Supplementary Material

Additional file 1: Table S1 Distribution of telomeric gene expression among the 12 non-cirrhotic and the 28 cirrhotic samples. Click here for file

Additional file 2: Table S2 Distribution of telomeric gene expression among the 28 cirrhotic and the 40 tumor samples. Click here for file

Additional file 3: Table S3 Distribution of telomeric gene expression among the 40 HCC and the 12 non-cirrhotic liver samples. Click here for file

Additional file 4: Table S4 Cause-specific distribution of telomere genes expression among the 28 cirrhotic liver samples. Click here for file

Additional file 5: Table S5 Cause-specific distribution of telomere genes expression among the 40 HCC samples. Click here for file
